# Role of heat shock protein 90 as an antiviral target for swine enteric coronaviruses

**DOI:** 10.1016/j.virusres.2023.199103

**Published:** 2023-03-28

**Authors:** Zhuangzhuang Zhao, Ya-Qing Zhang, Ling-Dong Xu, Lihua Xiao, Yaoyu Feng, Bin Wang, Yao-Wei Huang

**Affiliations:** a Department of Veterinary Medicine, Zhejiang University, Hangzhou, China; b Guangdong Laboratory for Lingnan Modern Agriculture, College of Veterinary Medicine, South China Agricultural University, Guangzhou, China

**Keywords:** Heat shock protein 90 (Hsp90), Coronavirus, Swine acute diarrhea syndrome coronavirus, Nucleoprotein, Gasdermin D, Pyroptosis

## Abstract

A variety of swine enteric coronaviruses (SECoVs) have emerged and are prevalent in pig populations, including porcine epidemic diarrhea virus (PEDV), transmissible gastroenteritis virus (TGEV), porcine deltacoronavirus (PDCoV), and swine acute diarrhea syndrome (SADS)-CoV, a newly identified bat-origin CoV with zoonotic potential. Unfortunately, available traditional, inactivated and attenuated SECoV vaccines are of limited efficacy against the variants currently circulating in most pig populations. In this study, we evaluated the role of host factor heat shock protein 90 (Hsp90) as an antiviral target against SECoVs, exemplified by SADS-CoV. Pharmacological inhibition of Hsp90 diminished SADS-CoV replication significantly in porcine and human cell lines, and also decreased replication of SADS-CoV in a porcine intestinal enteroid model. Further mechanistic experiments revealed that both porcine and human isoforms of Hsp90 interact with the SADS-CoV nucleocapsid (N) protein, and inhibition of Hsp90 resulted in autophagic degradation of N protein. Moreover, we linked Hsp90 to virus-induced cellular pyroptosis, as SADS-CoV was found to trigger caspase-1/gasdermin-d-mediated pyroptotic cell death, which was mitigated by inhibition of Hsp90. Finally, we demonstrated that Hsp90 also associated with N proteins and was involved in propagation of PEDV, PDCoV and TGEV. This study thus extends our understanding of immune responses to SADS-CoV infection and offers a new potential therapeutic option against four SECoVs.

## Highlights

• Targeting Hsp90 significantly reduced PEDV, PDCoV, TGEV or SADS-CoV infection in porcine cells or intestinal organoids.
• The N proteins of PEDV, PDCoV, TGEV or SADS-CoV are the clients of porcine Hsp90.
• Human Hsp90 interacts with the SADS-CoV N protein and prevents its autophagic degradation.
• SADS-CoV can trigger human caspase-1/GSDMD-mediated pyroptotic cell death.

## Graphical abstract

**Figure:**
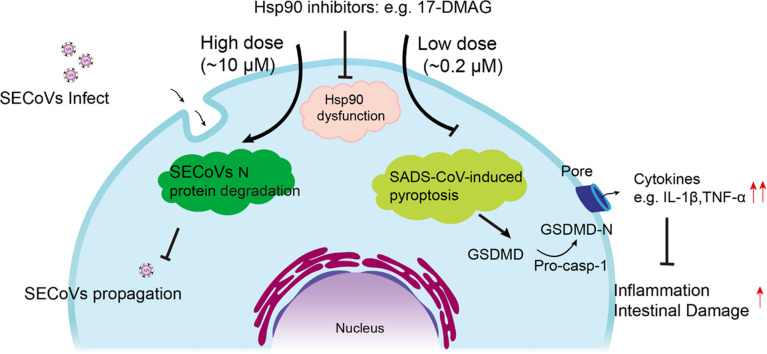

## 1 Introduction

Swine enteric coronaviruses (SECoVs), a group composed of porcine epidemic diarrhea virus (PEDV), transmissible gastroenteritis virus (TGEV), swine acute diarrhea syndrome (SADS)-CoV, and porcine deltacoronavirus (PDCoV), primarily cause enteric disease in pigs with similar clinical signs such as loss of appetite, diarrhea and vomiting, weight loss, lethargy, and death (Huang et al., 2013; Liu et al., 2021b; Wang et al., 2018a, 2019; Yang et al., 2020). Our group was among the first to isolate and characterize SADS-CoV after its discovery in 2017 (Pan et al., 2017), and the latest research has shown that it replicates effectively in human cells, suggesting a possible zoonotic potential (Edwards et al., 2020; Yang et al., 2019b). Unfortunately, available traditional, inactivated and attenuated SECoV vaccines are of limited efficacy against the variants currently circulating in most pig populations (Wang et al., 2019).

Since viruses depend on the host cell machinery for protein homeostasis, certain host factors may present ideal targets for novel broad-spectrum antiviral therapies. Heat shock protein 90 (Hsp90) is among the most abundantly expressed molecular chaperones in eukaryotic cells, and it participates in the stabilization and activation of more than 200 proteins, termed Hsp90 client proteins (Trepel et al., 2010). Mammalian Hsp90 has four isoforms: Two cytosolic isoforms (stress-inducible Hsp90α, and the constitutively expressed Hsp90β (Sreedhar et al., 2004)); and two non-cytosolic isoforms (TRAP1 and Grp94 located in mitochondria and endoplasmic reticulum, respectively (Chen et al., 2005)). Hsp90 is known to regulate the life cycle of diverse viruses through its chaperone functions on viral polymerase, capsid, and attachment proteins (Wang et al., 2017). Studies have shown that Hsp90 inhibitors could significantly decrease PDCoV infection in cultured cell lines (Zhao et al., 2021), but the underlying mechanism has not yet been demonstrated. In addition, it has not been studied whether inhibition of Hsp90 also limits infection by other SECoVs.

Recent studies have shown that in addition to decreasing CoV infection, Hsp90 inhibitors also downregulate expression of virus-induced inflammatory genes (Goswami et al., 2021; Wyler et al., 2021; Zhao et al., 2022b). Pyroptosis is a recently discovered inflammatory form of programmed cell death in which the sensor NLRP3 forms a functional inflammasome complex containing caspase-1 (Bader et al., 2022). Caspase-1 mediates the processing of IL-1β and IL-18 into their active forms, and cleavage of the pore-forming gasdermin D (GSDMD) induces pyroptotic cell death and the release of pro-inflammatory cytokines (Bergsbaken et al., 2009; Vande Walle and Lamkanfi, 2016). Emerging evidence suggests that CoVs are able to trigger pyroptosis (Ferreira et al., 2021; Ma et al., 2021; Sun et al., 2022), but it is unknown whether Hsp90 activity is involved. We hypothesized that Hsp90 activity is linked to virus-induced pyroptosis, resulting in a reduction of virus-mediated expression of pro-inflammatory cytokines.

Here we tested Hsp90 as an antiviral target against PEDV, TGEV, PDCoV and SADS-CoV infection, while also further dissecting the molecular mechanism by which Hsp90 inhibits SADS-CoV (as a representative) in porcine and human cells. We discovered that SADS-CoV could trigger pyroptosis, an effect that was mitigated by inhibition of Hsp90. This study thus extends our understanding of immune responses to SADS-CoV and provides a new potential therapeutic option against four SECoVs.

## 2 Materials and methods

### 2.1 Cell culture and virus

HEK293T, swine testicular (ST) cells, Caco-2 and Vero cells were maintained in Dulbecco's modified Eagle's medium (DMEM, Hyclone) supplemented with 10% (v/v) fetal bovine serum (FBS, Biological Industries). THP-1 cells, a human monocytic cell line, were cultured in RPMI 1640 medium (Gibco) containing 10% FBS (Gibco). Differentiation of THP-1 monocytes to macrophages was induced by 20 nM PMA (phorbol 12-myristate 13-acetate). All cells were cultured at 37 °C with 5% CO_2_.

The SADS-CoV isolate CH/GD-01/2017-p10 (GenBank accession no. MK977618) and a recombinant SADS-CoV expressing green fluorescent protein (GFP) (Yang et al., 2022b), the PEDV virulent strain ZJU/G2/2013 (GenBank accession no. KU558701), the TGEV Purdue strain and the PDCoV CH/Hunan/2014 strain (GenBank accession no. KY513724) were used in the study (Ji et al., 2018; Liang et al., 2023; Liu et al., 2021b; Qin et al., 2019; Yang et al., 2019a). SADS-CoV and PEDV viral titers were determined in Vero cells, whereas TGEV and PDCoV viral titers were determined in ST cells as described previously (Pan et al., 2017). Briefly, cultured cells were overlaid with viruses diluted in maintenance medium (MM) containing DMEM, 0.3% tryptose phosphate broth (TPB), and 1% penicillin/streptomycin. After 2 h of incubation at 37 °C, infected cells were washed with PBS and then cultured in MM.

### 2.2 Generation of Hsp90a-Ko and Hsp90b-Ko cell lines by CRISPR/Cas9

We used a CRISPR/Cas9 system (Komor et al., 2017) to generate Hsp90α^−/−^ (90α-KO) and Hsp90β^−/−^ (90β-KO) HEK293T cells. sgRNA-transfected cells were selected in the presence of puromycin for 48 h. After single cell clones were established, their genomic DNAs were sequenced to confirm the intended genetic disruptions.

### 2.3 Porcine intestinal enteroid (PIE) culture

PIEs were maintained and differentiated according to our protocol described elsewhere (Yang et al., 2022b). Briefly, porcine intestinal crypts isolated from piglets were resuspended with IntestiCult Organoid Growth Medium (STEM CELL) and Matrigel (Corning) and seeded in a 24-well plate for maturation. Mature enteroids were resuspended with IntestiCult Organoid Growth Medium and seeded into a Matrigel-coated 96-well plate. After 3 days of differentiation, the enteroid monolayers were ready for experimental use.

### 2.4 Plasmids

The complete coding regions of pig-Hsp90α (pig-Hsp90α; 733 aa in length; Gene ID: 397,028) and pig-Hsp90β (pig-Hsp90β; 803 aa in length; Gene ID: 397,191) were separately amplified by one-step RT-PCR using total RNAs extracted from ST cells, and subsequently cloned into a pCAGGS vector using KpnI and XhoI restriction sites and confirmed by DNA sequencing. The cloning primers are listed in Supplementary Table S1.

### 2.5 Reagents and antibodies

Tanespimycin (17-AAG) (cat. no. 75,747–14–7), alvespimycin (17-DMAG) HCl (cat. no. 467,214–21–7), HSP990 (NVP-HSP990) (cat. no. 934,343–74–5) and proteasome inhibitor MG-132 (cat. no. 1,211,877–36–9) were purchased from Selleck Chemicals. 3-methyladenine (HY-19,312) was purchased from MCE. Bafilomycin A1 (ab120497) was purchased from Abcam. Chloroquine (CAS 50–63–5), PMA (P1585), and nigericin (481,990) were purchased from Sigma. Transfection reagents (Lipofectamine 3000, Lipofectamine RNAiMAX) were purchased from Invitrogen. PI cell cytotoxicity assay kit (Cat: C2015M), Hoechst 33,342 (Cat: C1027), and DAPI (Cat: P0131) were purchased from Beyotime. CCK-8 Cell Counting Kit (A311–01) was purchased from Vazyme. CytoTox 96® Non-Radioactive Cytotoxicity Assay (G1780) was purchased from Promega.

Myc-tag (#2278), Hsp90α (#8165) rabbit monoclonal antibody, Hsp90β antibody (#5087), Hsp70 antibody (#4872), GSDMD antibody (#39,754) and anti-β-actin (#3700) mouse monoclonal antibody were purchased from Cell Signaling. GAPDH (#FD0063) antibody was purchased from Fudebio-tech. Caspase-1 antibody (ab207802) was purchased from Abcam. Mouse ANTI-FLAG® M2 monoclonal antibody (F3165) was purchased from Sigma. Alexa Fluor 488- or 647-conjugated goat anti-rabbit IgG and Dynabeads™ Protein G for immunoprecipitation (10004D) were purchased from Thermo Fisher Scientific. Anti-PDCoV-N and anti-PEDV-N monoclonal antibody were purchased from Medgene Labs (Brookings, SD, USA). Anti-TGEV-N and anti-SADS-CoV-N polyclonal antibody were generated in-house.

### 2.6 Immunofluorescence assay

ST or HEK293T cell lines including 90α-KO and 90β-KO were grown on slides, washed 2 times with PBS, fixed with 4% paraformaldehyde in PBS for 20 min and then permeabilized with 0.5% Triton X-100 for 10 min. Primary antibody (anti-myc-tag or anti-HSP90α/β, diluted 1:1000 in PBS) was added to cells and incubated for 1 h at 37 °C. Cells were washed twice with PBS and an Alexa Fluor 488- or 647-conjugated goat anti-rabbit IgG (Thermo Fisher Scientific, USA) was added as secondary antibody, followed by DAPI staining.

### 2.7 Quantitative real-time PCR

Total RNA was extracted from cells using Trizol (ThermoFisher Scientific, USA) following the manufacturer's instructions. Detection of mRNA expression levels by qRT-PCR was performed using SYBR green fluorescent dye (KAPA SYBR ® FAST ABI Prism ® qPCR kit, Kapa Biosystems Inc, MA, USA) with primers (see Supplementary Table S2).

Each of SECoV RNA titers was determined by one-step qRT-PCR (TOYOBO Co., LTD) targeting the N or M gene with the primers indicated in Table S2. Samples with a cycle threshold value <35 were considered positive based upon validation data using the RNA standards.

### 2.8 Immunoprecipitation (IP) assays and western blot

HEK293T cells seeded in a 6-well cell culture cluster were cotransfected with porcine or human-Hsp90α/β-flag and SADS-CoV-N plasmids (Liu et al., 2021a) or blank vector (as control) using Lipofectamine 3000 (Thermo Fisher). At 48 h post-transfection, cells were lysed with lysis buffer (25 mM Tris–HCl, 200 mM NaCl, 10 mM NaF, 1 mM Na_3_VO_4_, 25 mM β-glycerophosphate, 1% NP40, and protease cocktail [Biotool, Houston, TX]). 300 µL of supernatant was incubated with 4 µg of flag antibody overnight at 4 °C, followed by incubation with 30 µL of pre-washed Dynabeads™ Protein G at 4 °C for 12 h with mixing. After washing, the beads were eluted with 30 μL of elution buffer. The eluate was separated by 10% SDS PAGE and then applied to western blot analysis.

For cellular western blots, cells were lysed in lysis buffer, resolved by SDS-PAGE and transferred onto a polyvinylidene difluoride (PVDF) membrane that was subsequently blocked with Tris-buffered saline (TBS) containing 3% bovine serum albumin (BSA) overnight at 4 °C. Proteins were detected using the anti-myc antibody or anti-flag antibody at 1:1000 dilution, followed by incubation with horseradish peroxidase (HRP)-conjugated anti-rabbit IgG (1:5000 dilution; Thermo Fisher Scientific).

### 2.9 Transfection with siRNA

siRNA directed against pig-Hsp90α (siRNA-Hsp90α: 5′-GCGCUCCUUUCGACUUAUUTT-3′), pig-Hsp90β (siRNA-Hsp90β: 5′-GCAG

CAACAUAAACUCCUUTT-3′) or human-ATG7 (siRNA-ATG7: 5′-GCCGUGGA

AUUGAUGGUAU-3′) were purchased from RiboBio (Guangzhou, China). The siRNA transfections in ST cells were performed using Lipofectamine RNAiMAX transfection reagent (Thermo Fisher Scientific, USA) according to the manufacturer's instructions. At 48 h post-transfection, ST cells were infected with SADS-CoV at an MOI=5 or transfected with SADS-CoV-N plasmids.

### 2.10 Cell viability assay

ST or Caco-2 cells were seeded into 96-well tissue culture plates and incubated under standard conditions until the cells were 90% confluent. Then, the cell culture media was removed, and cells were incubated with 17-DMAG at concentrations of 0.5, 1, 2.5, 5 or 10 µM for 24 h, followed by detection of cell viability using a CCK-8 Cell Counting Kit. Optical density was measured at an absorbance of 450 nm. THP-1 cells were seeded into 24-well tissue culture plates and differentiated into macrophages, then supernatants were subjected to lactate dehydrogenase (LDH) assay to assess cell death using a CytoTox 96 non-radioactive cytotoxicity assay kit. Cell pellets were used to measure cell viability by PI Cell Cytotoxicity Assay Kit. Only dead cells were labeled with PI (red fluorescence).

### 2.11 ELISA

Cell culture supernatants were collected to measure levels of IL-1β (EH001–48; ExCell Bio) and TNF-α (EH009–48; ExCell Bio) using the respective ELISA kits according to the manufacturer's instructions.

### 2.12 Data analysis

Statistical analysis was performed using OriginPro 2017 software. Data are presented as mean ± standard error of the mean (SEM). Statistical comparisons were performed with one-way analysis of variance (ANOVA), using Bonferroni's post-hoc analysis or paired/unpaired Student's *t*-tests where appropriate. *P* values ≤ 0.05 were considered statistically significant.

## 3 Results

### 3.1 Pharmacological inhibition of Hsp90 reduces SADS-CoV production *in vitro*

**Fig. 1:**
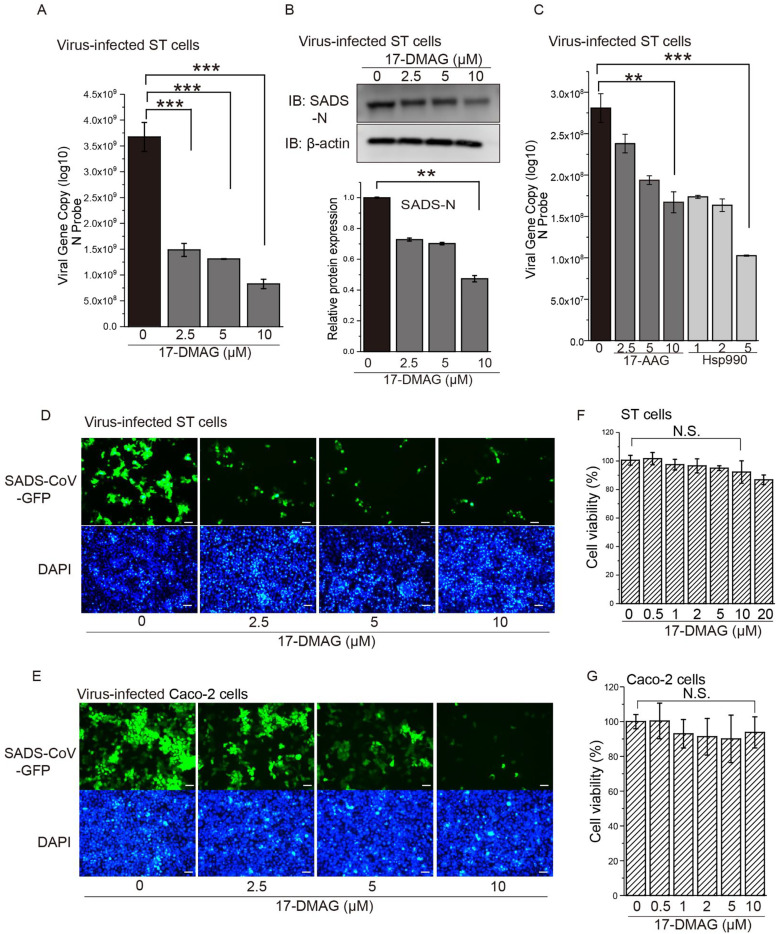
**17-DMAG inhibits SADS-CoV replication in pig cells and human cells.** ST cells were incubated with or without 10 μM 17-DMAG and infected with SADS-CoV at MOI=10 for 24 h. **(A)** Viral RNA was detected in SADS-CoV-infected ST cells by qRT-PCR. **(B)** Changes in the amount of SADS-CoV N protein in virus-infected ST cells by western blot. The relative level of proteins was quantified by immunoblot scanning and normalized with respect to the amount of β-actin (right panel). **(C)** ST cells were incubated with or without 17-AAG/ NVP-HSP990 and infected with SADS-CoV at MOI=1 for 24 h. Viral RNA was detected by qRT-PCR. **(D)** ST cells or **(E)** Caco-2 cells were incubated with or without 10 μM 17-DMAG and infected with SADS-CoV-GFP at MOI=10 for 24 h. Cells were stained with DAPI and observed under a fluorescence microscope (scale bar, 50 μm). Cytotoxicity was measured in **(F)** ST cells or **(G)** Caco-2 cells after 24 h treatment with 17-DMAG. Cell viability was determined and calculated as a percentage of viable cells after treatment with DMSO. *: *P* ≤ 0.05; **: *P* ≤ 0.01; ***: *P* ≤ 0.001; N.S.: not significant.

To investigate the role of Hsp90 in SADS-CoV propagation, we tested the effect of an Hsp90 inhibitor, 17-DMAG, in virus-infected ST cells. 17-DMAG treatment significantly decreased the SADS-CoV genome copy number (Fig. 1A) and viral N protein levels (Fig. 1B). The effects of Hsp90 inhibitors 17-AAG and NVP-HSP990 on SADS-CoV infection were also evaluated, showing that 17-AAG and NVP-HSP990 decreased SADS-CoV RNA production by qRT-PCR (Fig. 1C).

We also performed quantitative SADS-CoV infection analysis using a recombinant SADS-CoV expressing GFP (Yang et al., 2022b). Consistent with above, there was a marked decrease in GFP signal upon 17-DMAG treatment of SADS-CoV-GFP-infected ST cells compared to DMSO-treated controls (Fig. 1D). Since SADS-CoV has been shown to replicate effectively in human cells (Edwards et al., 2020; Yang et al., 2019b), we also looked at the inhibitory effect of 17-DMAG in a human enteric cell line, Caco-2. Again, there was a clear reduction of GFP signal in SADS-CoV-GFP-infected Caco-2 cells after 17-DMAG treatment compared to DMSO-treated controls (Fig. 1E). We checked the cytotoxicity of 17-DMAG in both ST and Caco-2 cells, confirming a lack of significant cell damage caused by any of the doses used (Fig. 1F,G).

### 3.2 Hsp90 inhibitor suppressed SADS-CoV replication *in vivo*

**Fig. 2:**
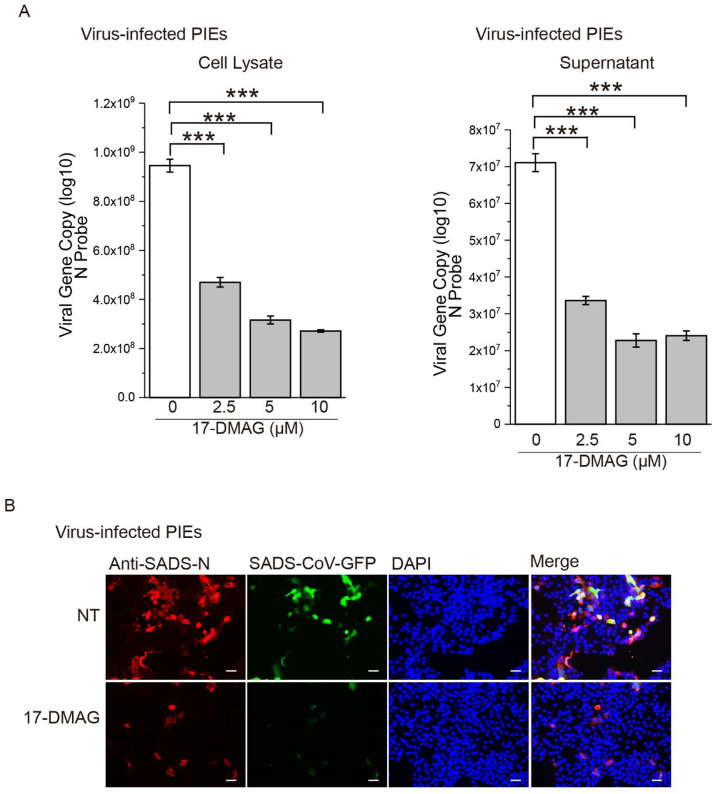
**17-DMAG inhibits SADS-CoV replication in PIEs.** PIEs were incubated with or without 10 μM 17-DMAG and infected with SADS-CoV-GFP at MOI=1 for 24 h. **(A)** Viral load was detected in PIEs lysate or supernatant by one-step qRT-PCR. **(B)** The infected PIEs were fixed, followed by immunofluorescence staining of SADS-CoV N protein (rad), nuclei were stained with DAPI (blue). SADS-CoV N protein (red) colocalized with SADS-CoV-GFP (green). Scale bar, 50 μm; ***: *P* ≤ 0.001.

Having shown that the Hsp90 inhibitor 17-DMAG decreases SADS-CoV replication *in vitro*, we wanted to determine whether it would be effective *in vivo*. We used porcine intestinal enteroids (PIEs) to mimic intestinal biology and physiology *in vivo*, a SADS-CoV infection model described in our previous study (Yang et al., 2022b). PIEs were generated from stem cells harvested from piglets, and differentiated enteroids are dissociated into two-dimensional enteroids monolayers for SADS-CoV infection. 17-DMAG treatment decreased the SADS-CoV viral load in the PIEs and in the culture medium (Fig. 2A). Consistent with viral loads, a marked decrease in GFP signal and viral N protein was observed in SADS-CoV-GFP-infected PIEs after 17-DMAG treatment compared to DMSO-treated controls (Fig. 2B).

### 3.3 Gene knockdown of porcine Hsp90 inhibits SADS-CoV infection

**Fig. 3:**
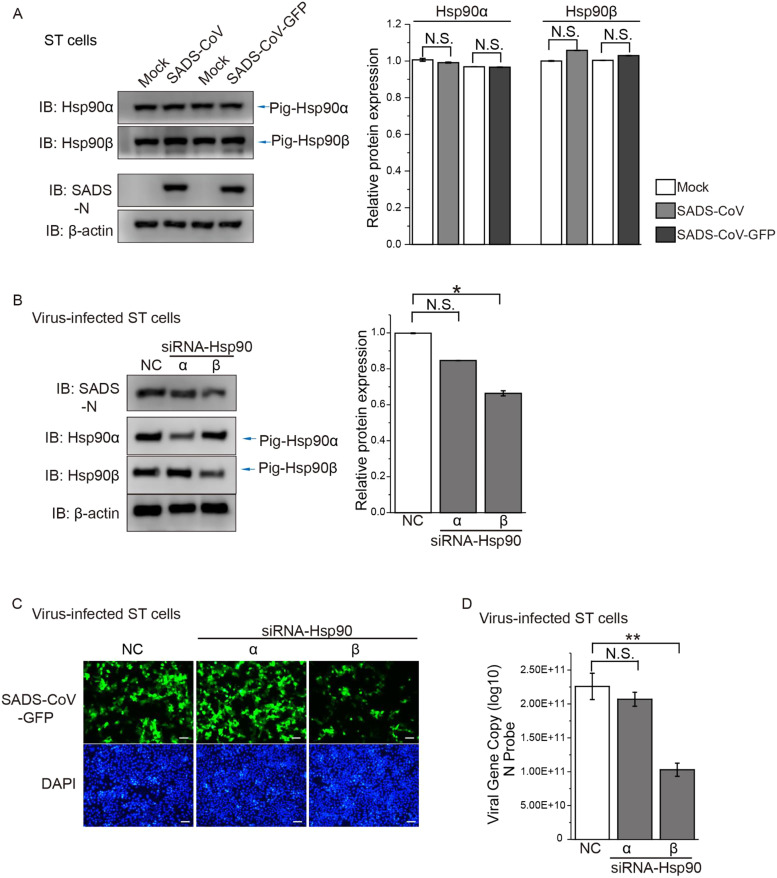
**Effect of porcine Hsp90 isoforms on SADS-CoV infection. (A)** ST cells were infected with SADS-CoV or SADS-CoV-GFP at MOI=10 for 24 h, and protein level of pig-Hsp90α/β were analyzed by western blot. **(B)** ST cells were transfected with siRNA-Hsp90α or siRNA-Hsp90β; the same amount of control siRNA was used as a negative control (NC). At 48 h post-transfection, cells were infected with MOI=10 of SADS-CoV for 24 h. SADS-CoV N protein level was quantified by immunoblot scanning and normalized with respect to the amount of β-actin (lower panel). **(C)** ST cells were transfected with siRNA-Hsp90α or Hsp90β. After 48 h of transfection, cells were infected separately with MOI=10 of SADS-CoV-GFP for 24 h. Cells were stained with DAPI to visualize nuclei (blue). **(D)** Viral RNA was detected in the cells by qRT-PCR; *: *P* ≤ 0.05; **: *P* ≤ 0.01; N.S.: not significant.

Given the two cytosolic isoforms of Hsp90 (Hsp90α and Hsp90β), we wanted to find out more about which one(s) play a role in SADS-CoV infection. First, we looked at the protein level of each isoform during SADS-CoV infection in ST cells. The expected band sizes of 91 kDa and 99 kDa were seen for porcine Hsp90α and Hsp90β, respectively (Fig. 3A). Image quantitation from three replicates showed that β-actin-normalized levels of Hsp90α and Hsp90β did not change significantly during SADS-CoV or SADS-CoV-GFP infection (Fig. 3A).

In order to determine the importance of individual isoforms, we used siRNA to knock down Hsp90α or Hsp90β in porcine cells, then infected them with SADS-CoV for 24 h. Treatment with siRNA effectively knocked down Hsp90α and Hsp90β in ST cells, and viral N protein production was significantly reduced after Hsp90β knockdown compared with the negative-control siRNA (Fig. 3B). Consistent with these results, there was a noticeable decrease in GFP signal following Hsp90β knockdown in SADS-CoV-GFP-infected ST cells, compared with both the negative control and Hsp90α knockdown (Fig. 3C). Moreover, viral genome copy number was significantly lower after Hsp90β knockdown in SADS-CoV-infected ST cells (Fig. 3D). Overall, siRNA depletion of Hsp90β, but not Hsp90α, significantly reduced SADS-CoV production in ST cells.

### 3.4 Porcine Hsp90 interacts with and maintains stability of SADS-CoV N protein

**Fig. 4:**
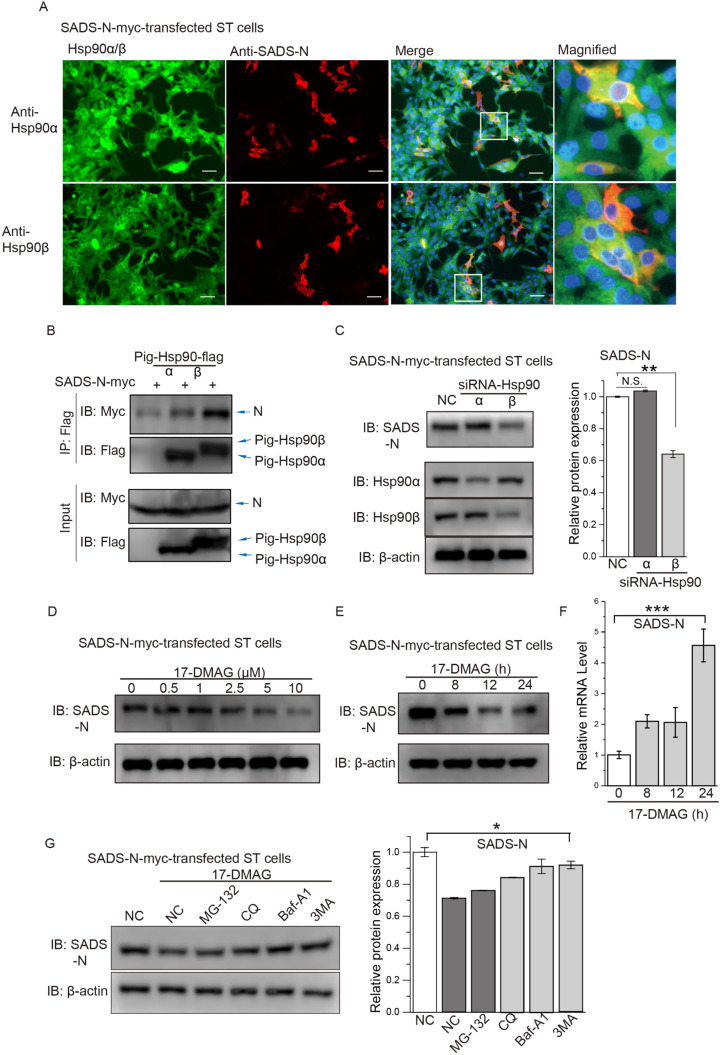
**Porcine Hsp90β interacts with SADS-CoV N protein. (A)** Colocalization of Hsp90α or Hsp90β with SADS-CoV N protein. ST cells were grown on coverslips and then transfected with a SADS-CoV N expression vector. At 24 h post-transfection (hpt), cells were fixed, permeabilized and incubated with primary antibodies directed against Hsp90α/β and viral N protein, followed by incubation with appropriate secondary antibodies. Cell nuclei were subsequently stained with DAPI (blue). **(B)** Co-immunoprecipitation (co-IP) assay analyzing the interaction between porcine Hsp90α/β isoforms with myc-tagged SADS-CoV N protein (N-myc) in HEK293T cells. Co-IP was performed using an anti-flag (F3165) antibody, and the co-IP protein complexes were screened by western blot using an anti-myc (71D10) antibody. **(C)** Effect of knockdown of two Hsp90 isoforms on viral N protein expression. ST cells were transfected with siRNA-Hsp90α or siRNA-Hsp90β. At 48 hpt, cells were transfected with a SADS-CoV-N expression vector for 24 h, and N protein was quantified by immunoblot scanning. **(D)** ST cells were transfected with an N-myc expression vector, and at 24 hpt, fresh medium was added containing the indicated concentrations of 17-DMAG. At 48 hpt, cell lysates were subjected to immunoblot with the indicated antibodies. β-actin was used to normalize band intensity, and the relative expression level of each viral protein was based on the levels of DMSO-treated cells. **(E)** ST cells were transfected with a SADS-CoV-N expression vector, and fresh medium containing 10 µM 17-DMAG was added at the indicated times. At 48 hpt, cell lysates were subjected to immunoblot with the indicated antibodies. β-actin was used to normalize band intensity, and the relative expression level of N protein was based on the levels of DMSO-treated cells. **(F)** Effect of 17-DMAG treatment on SADS-CoV N mRNA levels in cells from (E), as determined by qRT-PCR. **(G)** ST cells were transfected with SADS-CoV-N vectors, and at 24 hpt, cells were treated with 10 µM 17-DMAG for 16 h before being treated with DMSO or 10 µM 17-DMAG, together with 10 µM MG132, 5 mM 3-methyladenine (3MA), 50 µM chloroquine (CQ) or 1 µM bafilomycin A1 (Baf-A1) for 8 h. At 48 hpt, cell lysates were subjected to immunoblot with the indicated antibodies; ***: *P* ≤ 0.001.

Immunofluorescence was used to demonstrate cytoplasmic colocalization between both Hsp90α-flag and Hsp90β-flag and N-myc in contransfected ST cells (Fig. 4A). Coimmunoprecipitation (co-IP) assays were performed to detect SADS-CoV N protein interact with Hsp90. We investigated the interaction between myc-tagged SADS-CoV N protein (N-myc) and flag-tagged porcine Hsp90α (pig-Hsp90α-flag) or Hsp90β (pig-Hsp90β-flag) after cotransfection of HEK293T cells. Co-IP bands for both pig-Hsp90α and pig-Hsp90β were seen binding SADS-CoV N (Fig. 4B). Collectively, these results verify the interaction of the SADS-CoV N protein with both isoforms of porcine Hsp90.

Next, we knocked down Hsp90 in ST cells and transfected with N-myc expression vectors, showing that N protein was decreased in cells depleted of Hsp90β, whereas Hsp90α depletion showed negligible effect (Fig. 4C). Similar to Hsp90 depletion, pharmacological inhibition of Hsp90 on protein expression of N-myc were tested. ST cells were transfected with N-myc and then treated with a non-cytotoxic dose of 17-DMAG. There was a dose- and time-dependent decrease in N-myc expression following treatment with 17-DMAG (Fig. 4D-E). To rule out the inhibition of viral mRNA by 17-DMAG, we confirmed that N-myc mRNA levels were not reduced in N-myc-transfected ST cells treated with 17-DMAG (Fig. 4F).

When Hsp90 is inhibited, Hsp90 client proteins commonly undergo autophagic or proteasomal degradation (Mohl and Roy, 2019; Zhang et al., 2021). We wanted to identify which pathway was primarily responsible for the observed degradation of viral N protein upon inhibition of Hsp90. For this purpose, protein expression was measured in N-myc-transfected ST cells after treatment with specific inhibitors of the proteasome (MG132) or autophagy (3-methyladenine [3MA], chloroquine [CQ], or bafilomycin A1 [Baf-A1]). Hsp90-mediated degradation of viral N protein was blocked by CQ, Baf-A1 and 3MA (Fig. 4G), indicating that inhibition of Hsp90 leads to degradation of SADS-CoV N protein via the autophagy pathway.

### 3.5 Similar to porcine Hsp90, human Hsp90 also maintains stability of SADS-CoV N protein to support SADS-CoV infection

**Fig. 5:**
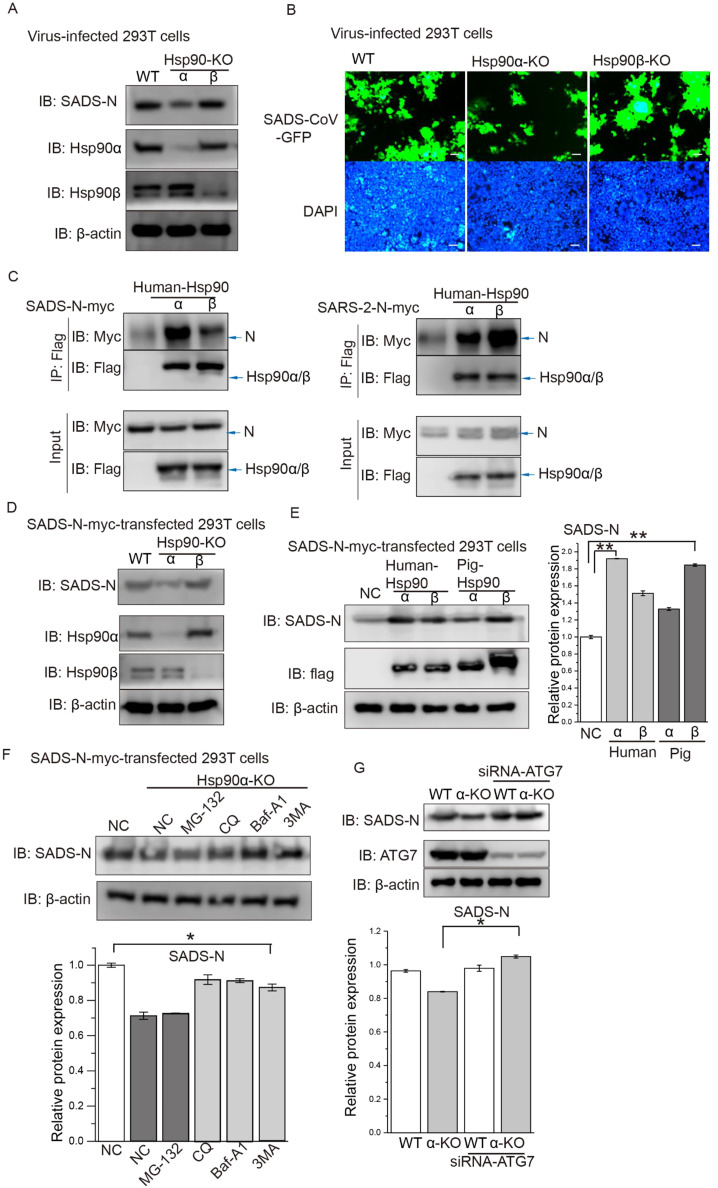
**Human Hsp90 activity is important for SADS-CoV replication by stabilizing N proteins. (A)** Wildtype (WT), Hsp90α-KO or Hsp90β-KO HEK293T cells were infected with MOI=10 of SADS-CoV-GFP for 24 h. The SADS-CoV N protein was analyzed by western blot. **(B)** Fluorescent microscopy of cells from (A), stained with DAPI for visualization of nuclei (blue). **(C)** WT HEK293T cells were transfected with myc-tagged SADS-CoV N (N-myc) and human-Hsp90β-flag or Hsp90α-flag vectors, with SARS-CoV-2 N protein as a positive control; total cellular proteins were extracted. Co-immunoprecipitation (co-IP) was performed using an anti-flag (F3165) antibody, and the co-IP protein complexes were screened by western blot analysis using an anti-myc (71D10) antibody. **(D)** WT, Hsp90α-KO or Hsp90β-KO HEK293T cells were transfected with an N-myc expression vector for 24 h, and the N protein level was quantified by immunoblot scanning. **(E)** Effect of overexpression of human or porcine isoforms of Hsp90 on SADS-CoV N protein expression in WT HEK293T cells. **(F)** WT or Hsp90α-KO HEK293T cells were transfected with an N-myc expression vector, and at 40 hpt the cells were then treated with 10 µM MG132, 5 mM 3MA, 50 µM CQ or 1 µM Baf-A1 for 8 h. At 48 hpt, cell lysates were subjected to immunoblot with the indicated antibodies. **(G)** WT or Hsp90α-KO HEK293T cells were transfected with siRNA-ATG7, and at 48 hpt the cells were transfected with an N-myc expression vector for 48 h. Cell lysates were subjected to immunoblot with the indicated antibodies.

First, we wanted to find out whether human Hsp90 play a role in SADS-CoV infection. CRISPR-Cas9 technology was used to create Hsp90α^−/−^ (90α-KO) or Hsp90β^−/−^ (90β-KO) mutant human HEK293T cell lines. After SADS-CoV infection, there was decreased in viral N protein produced in the 90α-KO cells compared with wildtype (WT) cells (Fig. 5A). Consistently, when using SADS-CoV-GFP, there was less GFP signal observed in infected 90α-KO cells compared to WT cells (Fig. 5B).

We further investigated the interaction between human Hsp90 and SADS-CoV N protein by contransfecting HEK293T cells with N-myc and human-Hsp90α-flag or Hsp90β-flag. Since studies have shown that Hsp90 is a host factor for severe acute respiratory syndrome (SARS)-CoV-2 (Li et al., 2020; Wyler et al., 2021), we used SARS-CoV-2-N-myc vector as a positive control. Co-IP bands for human Hsp90α and Hsp90β were seen for both SADS-CoV N and SARS-CoV-2 N proteins, and there was a noticeably greater interaction between SADS-CoV N protein and human Hsp90α, compared with Hsp90β (Fig. 5C).

Next, we compared the effect of Hsp90α/β knockout or overexpression in HEK293T cells on the production of N-myc. N-myc levels were decreased or increased by knockout (Fig. 5D) or overexpression (Fig. 5E) of human-Hsp90α in HEK293T cells, respectively, whereas human-Hsp90β seemed to have no effect. However, N-myc levels were increased by overexpression of pig-Hsp90β-flag, whereas pig-Hsp90α-flag overexpression had no effect (Fig. 5E), consistent with the knockdown results (Fig. 4C). Collectively, we can conclude that SADS-CoV N protein is a client of human Hsp90, and that its expression level is mainly regulated by Hsp90α rather than Hsp90β in human cells.

We further dissected the molecular mechanism by which Hsp90 regulates viral N protein in human cells. WT or 90α-KO HEK293T cells were transfected with N-myc, and then treated with the inhibitors MG132, 3MA, CQ, or Baf-A1. Hsp90-mediated degradation of N protein in HEK293T cells was blocked by CQ, Baf-A1 and 3MA (Fig. 5F). Autophagy related protein 7 (Atg7) is a critical component for the formation of autophagosome and required for autophagy processes. Knockdown of Atg7 by specific siRNA blocked Hsp90-mediated degradation of viral N protein (Fig. 5G), indicating that inhibition of Hsp90 leads to degradation of SADS-CoV N protein via the autophagy pathway.

### 3.6 Hsp90 is linked to virus-induced cellular pyroptosis

**Fig. 6:**
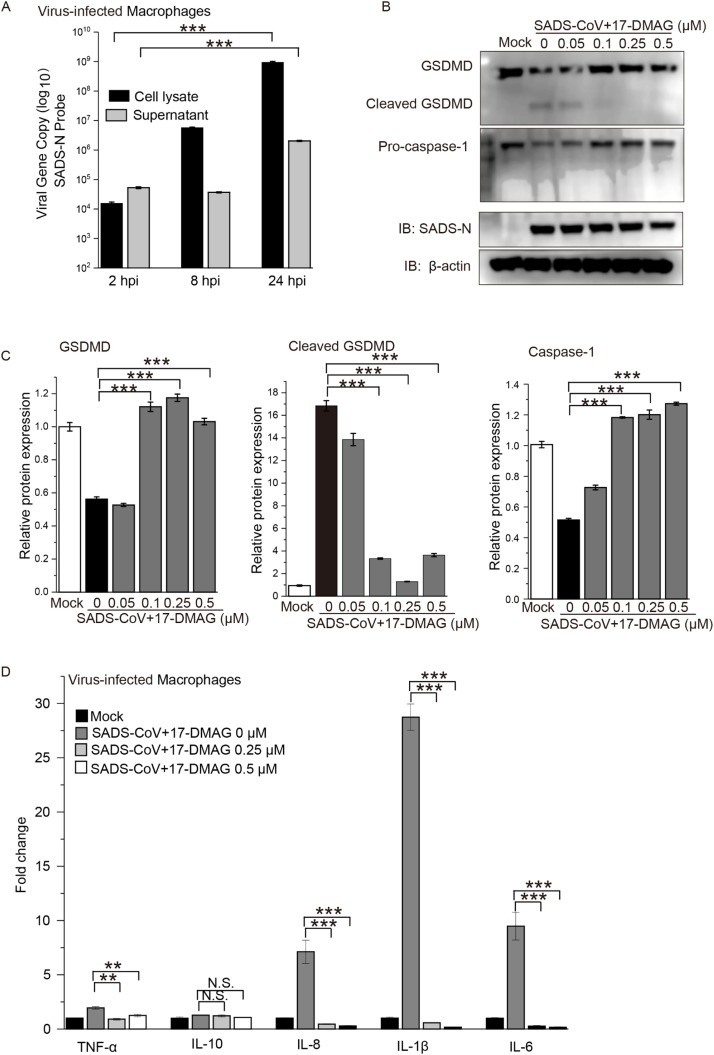
**SADS-CoV infection triggers GSDMD-mediated pyroptosis, which was reduced by inhibition of Hsp90. (A)** Macrophages were infected with SADS-CoV at MOI=10. Viral load was detected in cell lysate or supernatant by one-step qRT-PCR at the indicated hpi. **(B)** Macrophages were infected with SADS-CoV at MOI=10 for 24 h in the presence 17-DMAG. Cells were lysed and levels of pro-caspase-1, GSDMD, cleaved GSDMD, Hsp70, SADS-CoV N and β-actin were determined by western blot. **(C)** The protein level of GSDMD, cleaved GSDMD and pro-caspase-1 were quantified by immunoblot scanning and normalized with respect to β-actin. **(D)** qRT-PCR of TNF-α, IL-10, IL-8, IL-1β and IL-6 mRNA levels in Macrophages infected with SADS-CoV (MOI of 10) for 24 h in the presence 17-DMAG. The relative expression of target genes was normalized to GAPDH rRNA; **: *P* ≤ 0.01; ***: *P* ≤ 0.001; N.S.: not significant.

Previous studies have suggested that PEDV or TGEV are able to cleave GSDMD (Shi et al., 2022; Zhao et al., 2022a), which triggers pyroptosis, but it is unknown whether Hsp90 is linked to virus-induced cellular pyroptosis. We tested for the first time whether SADS-CoV could activate GSDMD-mediated pyroptosis in the human monocyte cell line THP-1, which is typically used for pyroptosis induction (Ferreira et al., 2021; Ma et al., 2021; Yang et al., 2022a). When THP-1 cell-derived M0 macrophages were infected with SADS-CoV at an MOI=10 for 24 h, a high abundance of viral RNA could be detected in the cells (Fig. 6A). Moreover, western blot revealed reduced pro-caspase-1 and cleaved GSDMD bands in SADS-CoV-infected macrophages, which were mitigated in intensity by treatment with 0.25 μM 17-DMAG (a low dose that did not affect viral replication according to N protein levels) (Fig. 6B-C). Moreover, we found that infection with SADS-CoV induced mRNA expression of inflammatory cytokines such as IL-6, IL-1β, and TNF-α, all of which could be prevented by 17-DMAG (Fig. 6D). These data demonstrate that SADS-CoV can induce caspase-1/GSDMD-mediated pyroptosis in macrophages that can be mitigated by inhibition of Hsp90.

### 3.7 Hsp90 inhibition suppresses cell death in SADS-CoV-infected macrophages

**Fig. 7:**
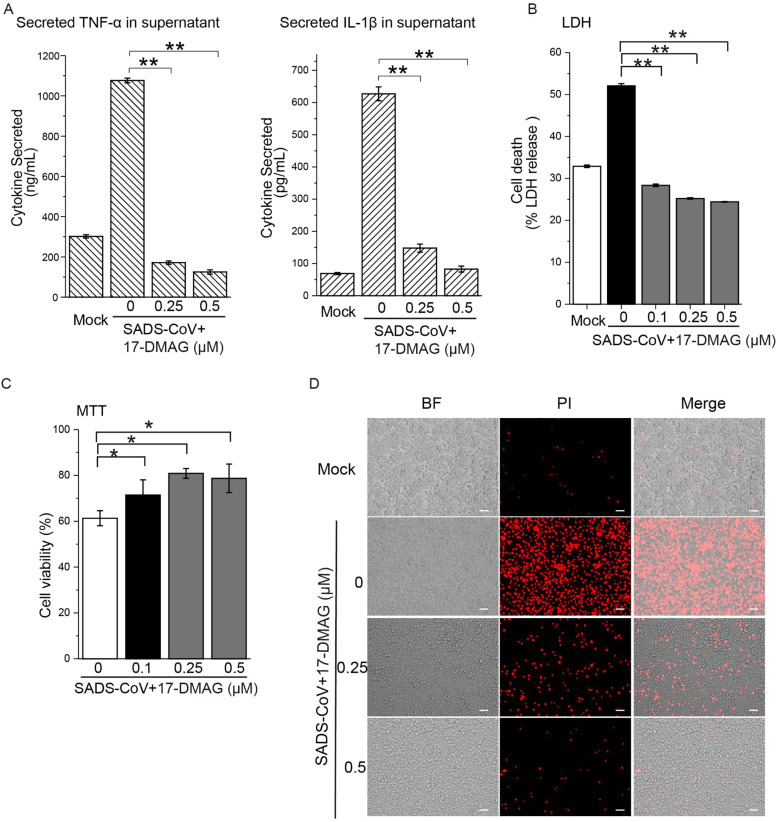
**Pyroptotic cell death triggered by SADS-CoV was mitigated by inhibition of Hsp90.** Macrophages were infected with SADS-CoV at MOI=10 for 24 h in the presence 17-DMAG. **(A)** ELISA was performed to determine IL-1β and TNF-α levels in cell supernatants. **(B)** Macrophages were infected with SADS-CoV in the presence 17-DMAG, then tested by LDH assay or MTT assay **(C)**, or stained with PI **(D)**; *: *P* ≤ 0.05; **: *P* ≤ 0.01.

Pyroptosis is a caspase-1-dependent event that leads to GSDMD pore formation (Frank and Vince, 2019) and release of pro-inflammatory cytokines (Man et al., 2017; Shi et al., 2017). We surveyed the spectrum of cytokines secreted by SADS-CoV-infected monocytes using an enzyme-linked immunosorbent assay (ELISA) array. We found supernatant levels of IL-1β and TNF-α in virus-infected cells were higher than in mock-infected cells, and 17-DMAG treatment reduced the secretion of IL-1β and TNF-α (Fig. 7A).

Cell death in SADS-CoV-infected macrophages was tested by lactate dehydrogenase (LDH) assay, cell cytotoxicity propidium iodide (PI) labeling assay, and MTT cell viability assays. SADS-CoV-infected macrophages underwent pyroptosis (greater cell death and lower cell viability), and treatment with 17-DMAG as low as 0.25 μM was sufficient to reduce the supernatant LDH activity (Fig. 7B), increase MTT cell viability (Fig. 7C) and decrease PI^+^ cells (Fig. 7D). These data suggest that although SADS-CoV infection could induce cell death in macrophages, 17-DMAG significantly improved cell viability.

### 3.8 Hsp90 also involved in propagation of other SECoVs

**Fig. 8:**
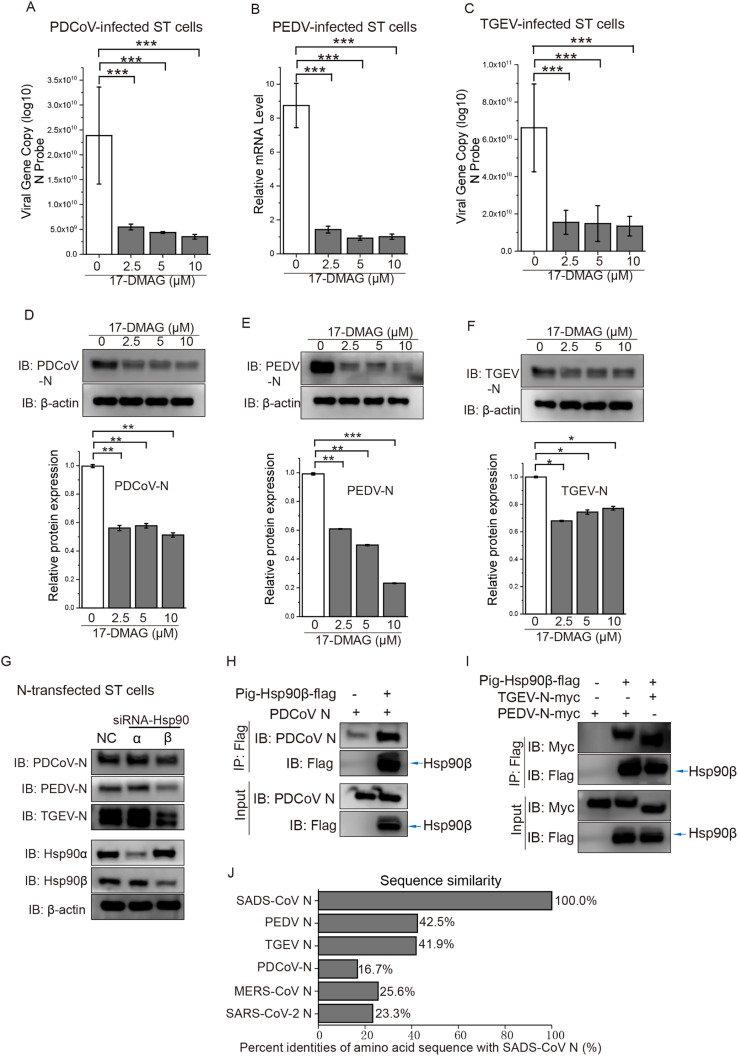
**Hsp90 also interacts with PDCoV/PEDV/TGEV N proteins and is involved in viral propagation. (A-F)** ST cells were incubated with or without 10 μM 17-DMAG and infected with viruses at MOI=10 for 24 h. Viral RNA was detected by qRT-PCR in ST cells infected by PDCoV **(A)**, PEDV **(B)** or TGEV **(C)**. Changes in the amount of viral N proteins were detected by western blot in ST cells infected by PDCoV **(D)**, PEDV **(E)** or TGEV **(F)**. Relative protein level was quantified by immunoblot scanning and normalized with respect to β-actin (lower panel); *: *P* ≤ 0.05; **: *P* ≤ 0.01; ***: *P* ≤ 0.001. **(G)** Effect of knockdown of two Hsp90 isoforms on PDCoV/PEDV/TGEV N protein expression. ST cells were transfected with siRNA-Hsp90α or siRNA-Hsp90β. At 24 hpt, cells were transfected with PDCoV/PEDV/TGEV N expression vectors for 36 h, and N protein was quantified by immunoblot scanning. **(H, I)** Co-IP assay analyzing the interaction between porcine Hsp90β isoforms with PDCoV/PEDV/TGEV N proteins. HEK293T cells were transfected with pig-Hsp90β-flag and PDCoV-N, PEDV N-myc or TGEV N-myc; total cellular proteins were extracted. Co-IP was performed using an anti-flag antibody. **(J)** Amino acid sequence similarity between the N proteins of SADS-CoV and other CoVs. The alignment was conducted by ClustalW, and the figure was generated by OriginPro 2017 software according to the similarity calculated by DNASTAR MegAlign.

We next analyzed whether the involvement of Hsp90 in SADS-CoV infection was common among other SECoVs. ST cells were infected with PEDV, PDCoV or TGEV at MOI=10, and cultured in the presence of various 17-DMAG concentrations for 24 h. Hsp90 inhibition resulted in significantly lower genome copy and viral N protein levels (Fig. 8). Further analyses into the mechanism of action of the Hsp90 inhibitors revealed that the effects of Hsp90 on PEDV/PDCoV/TGEV N proteins were similar to SADS-CoV. We knocked down Hsp90 in ST cells and transfected with PEDV/PDCoV/TGEV N expression vectors, showing that N protein was decreased in cells depleted of Hsp90β, whereas Hsp90α depletion showed a negligible effect (Fig. 8G). Co-IP assays were performed to detect interaction of SECoV N proteins with Hsp90β. We investigated the interaction between flag-tagged porcine Hsp90β (pig-Hsp90β-flag) and myc-tagged PEDV (PEDV N-myc), TGEV N (TGEV N-myc) or untagged PDCoV N (PDCoV-N) after cotransfection of HEK293T cells. Co-IP bands for pig-Hsp90β were seen binding PDCoV N, PEDV N and TGEV N proteins (Fig. 8H,I). Thus, Hsp90β could interact with all 4 SECoV N proteins, although the amino acid similarity between them was only around 16.7–42.5% (Fig. 8J) (Liu et al., 2021a).

## 4 Discussion

Some CoVs are a threat to human health including SARS-CoV, Middle East respiratory syndrome (MERS)-CoV, and SARS-CoV-2. Other CoVs infect pigs and are threats to the global swine industry and public health, including the four SECoVs that cause porcine diarrhea (Huang et al., 2013; Wang et al., 2019; Yang et al., 2020). SECoVs are a major concern since there currently are no effective treatments. Hsp90 is a host factor that is required for replication by many viruses from different taxonomic groups (Katoh et al., 2017; Mohl and Roy, 2019; Newman et al., 2018; Sakata et al., 2019; Wang et al., 2017, 2018b; Zhang et al., 2020). In the present study, we evaluated the host factor Hsp90 as a target for antiviral therapy against SECoVs *in vitro*. Our data demonstrate that the Hsp90 inhibitor 17-DMAG significantly decreased infection by SECoVs, and we conclude that all 4 SECoV N proteins are novel clients of Hsp90, because Hsp90 physically interacts with N protein and is required for its stability.

17-DMAG is a derivative of geldanamycin in the clinic, which inhibits the activity of Grp94 and cytoplasmic Hsp90 isoforms (Gorska et al., 2012; Taldone et al., 2013). Of four SECoVs, the newly identified SADS-CoV was selected for detailed study due to the significance of its zoonotic potential (Yang et al., 2020) and interspecies transmission (Mei et al., 2022). A limitation of the current study is that we only explored the effect of cytoplasmic Hsp90 isoforms on SADS-CoV infection. However, our data clearly demonstrate that cytoplasmic Hsp90 isoforms play key roles in SADS-CoV infection. siRNA depletion of porcine Hsp90β, but not porcine Hsp90α, significantly reduced SADS-CoV production in ST cells, which was evidenced by the reduction of viral loads, viral N protein and GFP signal of SADS-CoV-GFP virus (Fig. 3B-D). In addition, knockout of Hsp90α or Hsp90β in human cells reduced the infection of SADS-CoV (Fig. 5A-B).

As mentioned before, SADS-CoV is able to infect a variety of human cells, which may pose a threat to human health (Yang et al., 2019b). We further investigated the molecular mechanism of Hsp90 action on SADS-CoV propagation in porcine and human cells. Since previous reports have shown that Hsp90 interacts with MERS-CoV N protein (Li et al., 2020), we hypothesized that Hsp90 is a broad-spectrum chaperone for CoV N proteins. A co-IP assay revealed that both porcine and human Hsp90 could interact with the SADS-CoV N protein, and colocalization was seen in the cells (Figs. 4A-B, 5C). Although the amino acid similarity between the N proteins of SADS-CoV and MERS-CoV is only around 25.6% (Fig. 8J), Hsp90 is believed to recognize a metastable structural element found in client proteins rather than a primary amino acid motif (Geller et al., 2012). Indeed, our data demonstrate that Hsp90 also interacts with the N protein of PDCoV, PEDV and TGEV (Fig. 8H,I), which have 16.7–42.5% amino acid similarity with the N protein of SADS-CoV (Fig. 8J). Similar to other Hsp90 client proteins, both porcine and human Hsp90 participated in the stabilization of SADS-CoV N protein, pharmacological inhibition of Hsp90 also decreased the expression of transfected N protein. Furthermore, we found Hsp90 dysfunction-induced degradation of N protein was alleviated by CQ, Baf-A1 and 3MA, suggesting the degradation occurs via autophagy. Collectively, our study provides a mechanism whereby Hsp90 chaperones SADS-CoV N protein, and inactivated Hsp90 leads to a diminished viral propagation in cell lines and PIEs.

In addition, we noted a difference in the different isoforms of Hsp90 used to protect N from autophagic degradation in pig and human cells. Porcine Hsp90β interacted more with SADS-CoV N protein compared with Hsp90α, and it was key to maintaining the stability of SADS-CoV N protein, as N protein was significantly increased or decreased by overexpression or genetic depletion of Hsp90β (Figs. 3B, 5E). In human cells, Hsp90α interacted more with SADS-CoV N protein compared with Hsp90β. Hsp90α functions to maintain the stability of SADS-CoV N protein, as N protein were significantly increased or decreased by overexpression or genetic depletion of human Hsp90α (Figs. 4C, 5E). There has been little research on porcine Hsp90 proteins to analyze their differences with human Hsp90 proteins, although the relatively close genetic relationship between pigs and humans make their proteins similar, with 88.4% amino acid similarity between pig Hsp90α protein and human Hsp90α protein, 51% amino acid similarity between pig Hsp90β protein and human Hsp90β. Nevertheless, the relationship between porcine Hsp90 proteins and human Hsp90 proteins definitely requires further investigation.

Pyroptosis is a recently discovered inflammatory form of programmed cell death, mainly triggered by intracellular pathogens (Man et al., 2017), and it has been observed in the infection scenario of several CoVs. For the first time, we found that SADS-CoV can trigger caspase-1/GSDMD-mediated pyroptotic cell death. Inhibition of Hsp90 has been previously shown to downregulate the expression of virus-induced pro-inflammatory cytokines (Wyler et al., 2021; Zhao et al., 2022b), but whether Hsp90 is linked to virus-induced cellular pyroptosis is still unknown. We found that an extremely low-dose of 17-DMAG could greatly reduce virus-induced pyroptosis including inhibition of cell death and cleavage of GSDMD, and reducing the secretion of IL-1β and TNF-α in SADS-CoV-infected macrophages. 17-DMAG-mediated repression did not decrease the accumulation of viral proteins, as low-dose 17-DMAG did not affect SADS-CoV replication (Fig. 6B). A limitation of these studies is that 17-DMAG only repressed virus-induced pyroptosis in human cells. In ST cells, even in PIEs, SADS-CoV hardly induced cellular pyroptosis (defined by GSDMD cleavage or pro-inflammatory cytokine expression; data not shown). This result is similar to a study showing no significant change in the expression of TNF-α in PIEs following SADS-CoV infection (Yang et al., 2022b).

In recent years multiple novel pathways of programmed cell death were discovered, including necroptosis, apoptosis, autophagy, pyroptosis, and others (Bertheloot et al., 2021). Hsp90 is closely concerned with programmed cell death by participating in the degradation and activation of clients related to programmed cell death (Peng et al., 2022), including receptor-interacting serine/threonine kinase (RIP) 1 in necroptosis (Chen et al., 2012; Hu et al., 2020), Beclin-1/Bcl-2 in apoptosis and autophagy (Hasan et al., 2020), cellular-FLICE inhibitory protein (FLIP) in apoptosis (Wang et al., 2012), unc-51 like autophagy activating kinase 1 (ULK1) in autophagy (Joo et al., 2011), glutathione peroxidase 4 (GPX4) in necroptosis (Peng et al., 2022; Wu et al., 2019). And among four Hsp90 isoforms, TRAP1 and Grp94 are a cellular regulator of apoptosis (Montesano Gesualdi et al., 2007; Pan et al., 2009), Hsp90α and Hsp90β are also essential for apoptosis (Fritsch et al., 2016; Solier et al., 2012). Pyroptosis is a recently discovered inflammatory form of programmed cell death, The Hsp90 inhibitor geldanamycin is a recently discovered drug that was able to rescue THP-1 cells from lipopolysaccharide (LPS)-induced pyroptosis, achieved through the misfolding and degradation of NLRP3 (Mayor et al., 2007; Zhou et al., 2020). Consistently, our data show that the Hsp90 inhibitor 17-DMAG reduced virus-induced pyroptosis. However, the current studies mainly focus on the pan-inhibition of pyroptosis by Hsp90 inhibitors and was not able to determine which Hsp90 isoform was important for pyroptosis, pyroptosis regulated by different Hsp90 isoforms remain to be further investigated.

**Fig. 9:**
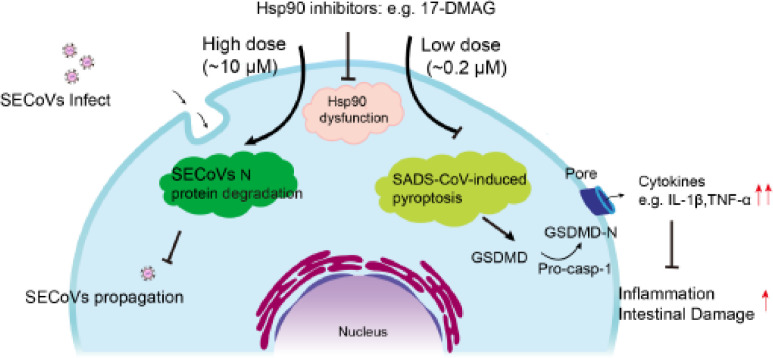
**Host Hsp90 is an antiviral target of swine enteric CoVs.** Hsp90 regulates swine enteric CoV infection by chaperoning and preventing degradation of viral N protein. High-dose 17-DMAG (∼10 µM) was more likely to result in greater N protein degradation and SADS-CoV clearance. Low-dose 17-DMAG (∼0.2 µM) delayed SADS-CoV-induced pyroptosis. SADS-CoV infection promoted cleavage activation of GSDMD, but targeting Hsp90 inhibited this process, which may work to reduce the subsequent hyperinflammation and pyroptosis that contributes to severe disease.

In conclusion, our study evaluated the host factor Hsp90 as a novel strategy against SECoVs. On one hand, both porcine and human Hsp90 serve as a chaperone for N protein of SADS-CoV and other SECoVs, preventing its degradation. On the other hand, low-dose 17-DMAG strongly reduced cell pyroptosis in SADS-CoV-infected macrophages, thus Hsp90 is expected to play a role in the inflammatory immune response in the cells when damaged by SADS-CoV (Fig. 9). These new insights into the host-virus interaction of SADS-CoV provide a theoretical basis for novel therapies against multiple SECoVs.

## CRediT authorship contribution statement

**Zhuangzhuang Zhao:** Conceptualization, Data curation, Formal analysis, Methodology, Validation, Writing – original draft. **Ya-Qing Zhang:** Data curation, Formal analysis, Methodology, Validation. **Ling-Dong Xu:** Data curation, Formal analysis, Methodology, Validation. **Lihua Xiao:** Resources. **Yaoyu Feng:** Resources. **Bin Wang:** Data curation, Formal analysis, Methodology, Validation, Resources. **Yao-Wei Huang:** Conceptualization, Methodology, Visualization, Project administration, Resources, Supervision, Funding acquisition, Writing – review & editing.

## Declaration of interests

The authors declare that they have no known competing financial interests or personal relationships that could have appeared to influence the work reported in this paper.

## Data Availability

Data will be made available on request.

## Appendix Supplementary materials

Image, application 1
