# Supplementary material for: Role of heat shock protein 90 as an antiviral target for swine enteric coronaviruses
Source: Virus Res. 2023 Mar 28;329:199103. doi: 10.1016/j.virusres.2023.199103 (PMC10194284; doi:10.1016/j.virusres.2023.199103)
Supplement: Supplementary file 1 [file mmc1.docx]

Table S1. Primers used in plasmid construction.

| Gene Primers (5’-3’) for cloning into PCAGGS-Flag |  |
| --- | --- |
| Pig-Hsp90β FP1: AAGAATTCGAGCTCATCGATGCATGGTACCATGAGGGCCCTGTGGGTGCTGGGCCTGTGC  RP1: TTTATCATCATCATCTTTGTAATCCAGTTCATCTTTTTCAGCTGTAGATGTCTCTGATGT  RP2: GGCAGAGGGAAAAAGATCTGCTAGCTCGAGTCATTTATCATCATCATCTTTGTAA | |
| Pig-Hsp90α FP1: AATTCGAGCTCATCGATGCATGGTACCATGCCCGAGGAAACCCAGACCCAAGACCAG |  |
| RP1: GACGACACGTCCCGCATGGAGGAAGTCGATGATTACAAAGAT |  |
|  |  |

Table S2. Oligonucleotides used for RT-qPCR

| Gene Primers (5’-3’) |  |
| --- | --- |
| TGEV-N-qPCR-F GAGCTAGAAGCAGTTCAG  TGEV-N-qPCR-R CACAGATGGAACACATTC  TGEV-N-probe FAM-TAGCACCACGACTACCAAGC-TAMRA  PDCoV-M-qPCR-F ATTGACCACATGGCTCCAA  PDCoV-M-qPCR-R CAGCTCTTGCCCATGTAGCTT  PDCoV-M-probe FAM-CACACCAGTCGTTAAGCATGGCAAGCT-TAMRA  SADS-CoV-N-qPCR-F CTAAAACTAGCCCCACAGGTC | |
| SADS-CoV-N-qPCR-R TGATTGCGAGAACGAGACTG  SADS-CoV-N-probe FAM-GAAACCCAAACTGAGGTGTAGCAGG-TAMRA  PEDV-N-qPCR-F AATAAAGGAAATAAGGACCAGCA  PEDV-N-qPCR-R CAACCCAGAAAACACCCTCAGTA  Human TNFα FP1: CCAGACCAAGGTCAACCTCC  RP2: CAGACTCGGCAAAGTCGAGA  Human IL-6 FP1: AGGAGACTTGCCTGGTGAAA  RP2: CAGGGGTGGTTATTGCATCT  Human IL-8 FP1: TTGGCAGCCTTCCTGATTTC  RP2: CTTTAGCACTCCTTGGCAAAAC  Human IL-10 FP1: GATTTTAATAAGCTCCAAGACCAAGGT  RP2: CTTCTATGCAGTTGATGAAGATGTCAA  Human IL-1β FP1: ATGATGGCTTATTACAGTGGCAA  RP2: GTCGGAGATTCGTAGCTGGA  Human Gapdh FP1: GGAGTCAACGGATTTGGT  RP2: TGATGGGATTTCCATTG |  |
| FR: Forward Primer; RP: Reverse Primer; nt: nucleotides; |  |
